# Methods of Improving Speech Intelligibility for Listeners with Hearing Resolution Deficit

**DOI:** 10.1186/1746-1596-7-129

**Published:** 2012-09-25

**Authors:** Adam Kupryjanow, Andrzej Czyzewski

**Affiliations:** 1 Multimedia Systems Department, Faculty of Electronics, Telecommunications and Informatics, Gdansk University of Technology, Gdansk, Poland

## Abstract

**Abstract:**

Methods developed for real-time time scale modification (TSM) of speech signal are presented. They are based on the non-uniform, speech rate depended SOLA algorithm (Synchronous Overlap and Add). Influence of the proposed method on the intelligibility of speech was investigated for two separate groups of listeners, i.e. hearing impaired children and elderly listeners. It was shown that for the speech with average rate equal to or higher than 6.48 vowels/s, all of the proposed methods have statistically significant impact on the improvement of speech intelligibility for hearing impaired children with reduced hearing resolution and one of the proposed methods significantly improves comprehension of speech in the group of elderly listeners with reduced hearing resolution.

**Virtual slides:**

http://www.diagnosticpathology.diagnomx.eu/vs/2065486371761991

## Background

Time scale modification algorithms have been widely used for supporting various types of speech perception disorders. The main fields of TSM application are: Language Learning Impairment (LLI) [1-3], Second-Language Learning [4,5], Central Auditory Processing Disorders (CAPD) [6-9], verbal apraxia [10] and aphasia [9]. Despite the large number of works that are devoted to the influence of the time-expanded speech on several disorders, there are still deficiencies in this area. For example verbal apraxia and aphasia were only mentioned by Coyle [10] and Nejime [8] but evaluation of the TSM methods with such a group of subjects was not performed. Serve-to-profoundly hearing impaired children were examined by Uchanski et al. [11], but in that research, analysis of the hearing resolution impairment caused by the CAPD was not investigated.

In this work, three methods for real-time TSM, dedicated for listeners with hearing resolution deficit are presented. Effectiveness of these methods was examined for two different age groups of listeners: the hearing impaired children and the elderly persons with presbycusis. The latter group was chosen based on the assumption that main difficulties in speech comprehension in elderly people are associated with central auditory processing aspects of hearing [12,13]. The same hypothesis was the principle of speech modification methods proposed by Nakamura [6] and Nejime [9]. The former was selected because of the lack of research results in the area of analysis of relationship between the CAPD and time-expansion of speech. Uchanski [11] has shown that there is no statistically important impact on the speech recognition rate for the group of hearing impaired children, but in that research, hearing resolution deficit of speech perception (related to the CAPD) was not investigated. Therefore, in this paper hearing resolution of the listener was analysed in accordance with the time-expanded speech intelligibility in order to investigate the relationship between the hearing resolution deficit and speech intelligibility. We have made a hypothesis that for listeners with a reduced hearing resolution, time expansion of a fast rate speech using the proposed real-time TSM methods significantly improves speech perception.

The proposed TSM methods were designed in such a way that they could work in real-time on mobile devices. Applications of these methods may be the same as those proposed by Nakamura [6] (i.e. stretching the speech during television news), or by Nejime [9] (i.e. a portable device with built-in microphone and binaural headphones). Moreover, we propose a new application of this method, i.e. stretching the speech during the phone call (an algorithm implemented on a mobile phone). We used a smartphone as a mobile platform for implementation of the proposed methods. Tests of the capabilities of the mobile phone implementation were performed and the performance results were described in earlier papers [14-16]. In this work, influence of the proposed methods on the comprehension of speech was investigated.

The outline of the paper is as follows. In Section II, the proposed TSM methods are described. In Section III, usability of these methods is investigated and the results of speech comprehension tests for both listener groups are presented. The obtained results are discussed in Section IV and concluded in the last part of the paper.

## Methods

The proposed TSM methods were designed in order to modify, in real-time, a speech signal captured by the microphone located near the speaker’s mouth or the speech signal sent from a device (e.g. a cellphone, TV etc.). Three different TSM methods for the real-time speech stretching are proposed: an uniform real-time TSM (algorithm A) described by authors in an earlier paper [16], and two non-uniform real-time TSMs. One of the non-uniform TSMs was described in a conference paper (algorithm B) [14], while the second method provides a novel solution (algorithm C).

All the proposed TSM methods are based on the assumption that the input signal contains redundant information, i.e. silence passages (pauses between words, sentences, speeches) and prolonged vowels. These parts of the signal may be removed or at least they should not be stretched. This approach allows saving extra time in which the stretched speech could be presented.

**Figure 1:**
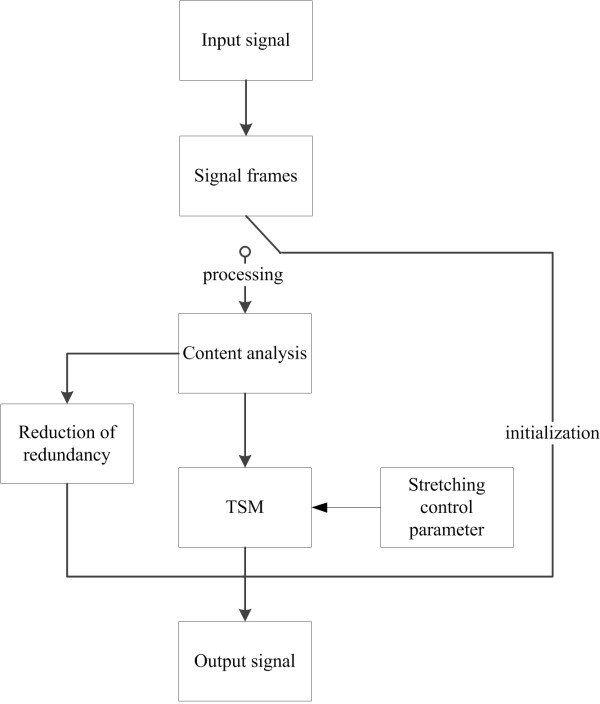
Block diagram of the proposed real-time TSM method.

In addition, as it was postulated by Coyle [10], Chu [17] and Demol [18], the non-uniform TSM was used for methods B and C, in order to obtain a high-quality and natural-sounding stretched speech. Non-uniform TSM is performed using various values of scaling factors for different speech units i.e. vowels, consonants and phone transitions. Scaling factors are chosen in a way that preserves the natural prosody, i.e. vowels are stretched with higher factors than for consonant, while phone transitions remain intact. Depending on the input speech rate, the signal is modified with different scaling factors. The way in which scaling factors are selected is related to the type of TSM method. The procedure of factors adjustment is described in the next sections. The block diagram of the proposed real-time TSM method is shown in Figure 1.

All of the algorithms used in the content analysis block were described in details in earlier papers [14-16], thus they will not be discussed here. The content analysis consists of: voice activity detection algorithm (VAD), vowel detection algorithm, rate of speech (ROS) estimation, stutter detection and phone transitions detection. As the core of the TSM, a SOLA (Synchronous Overlap and Add) algorithm was used. It was shown that this algorithm ensures high quality of the stretched speech and low computational complexity [19]. Moreover, SOLA method uses constant values of the analysis time shift and constant length of the analysis time frame. This fact allows for integrating the content analysis algorithms with the TSM procedure in a natural way, i.e. every time a frame of the input signal is analyzed in order to identify its content. Subsequently, based on results provided by the content analysis algorithms, the TSM procedure is performed. The parameter determining the amount of time-scale modification is called a scale factor *α*(*t*). It is defined by the equation (1):

(1) $$\alpha \left(t\right)=\frac{Ss}{Sa}$$

where *Sa* is the time shift of the frame used during the analysis step, *Ss* is the time shift of the frame used during the synthesis step. If the value of α(*t*) is greater than 1, the input signal will be stretched, if α(*t*) is lower than 1, the signal will be shortened; for α(*t*) equal to 1, the time scale modification will not be performed. Since the TSM will be performed only in order to expand the time of the input signal, *α*(*t*) will take values equal or higher than 1.

### Uniform speech stretching (method A)

In this method, a speech signal is stretched using constant values of the scaling factor. Input signal is time-extended only when the voice is detected by the VAD and vowel prolongation was not observed by the vowels detector. Despite the fact that the input signal is non-uniformly time scaled (silence and speech passages are modified using different stretching factor values), the speech signal is modified uniformly (with a single value of the stretching factor). The stretching procedure is controlled by the α_d_ parameter (representing desired scaling factor). The value of α_d_ should be specified (in the experiment it was set to a constant value equal to 1.5). Additionally, elimination of redundancy in the input signal is performed by replacing intervals of silence longer than 200 ms with the time-expanded speech.

### Non-uniform TSM controlled by a scaling factor (method B)

The second method of time-expansion of speech signals is performed using the same principles as in the method A, but additionally, the scaling factor values may vary depending on the input signal content and the ROS. Values of *α*(*t*) used in this method are presented in Table 1. The symbol α_d_ stands for the value of the scaling factor specified by the user. The rate of speech is estimated based on the analysis of vowels positions. Speech with the rate higher than or equal to 5.16 vowels/s is marked as fast. Selection of this threshold was based on the manually labeled utterance rates (slow, fast), where the average value and standard deviation of ROS obtained from all the recordings in the database, were calculated [14]. Whenever the fast spoken speech is detected, higher values of *α*(*t*) are used, and for speech with a normal rate, these values are reduced. Two additional restrictions were added to ensure that vowels will be stretched using values of *α*(*t*) not lower than for consonants: for slow speech, if the calculated value of *α*(*t*) is lower than 1, it is set to 1, and for fast speech, if the calculated value of *α*(*t*) is lower than 1.1, it is set to 1.1. The important is also fact that only not for all silence passages *α*(*t*) is defined because some of them are removed to ensure the synchronization between the input and output signal.

**Table 1:** Values of the scaling factor used in method B

**ROS [vowels/s]**	**α**			
	**vowels**	**consonants**	**silence**	**phones transients**
≥5,16	α_d_	0.8α_d_	1	1
<5,16	0.95α_d_	1.1	1	1

### Non-uniform TSM controlled by estimated ROS (method C)

Two methods presented above use the scaling factor as the control value of the output speech rate. This is not a natural way of specifying the speech rate, since for the same values of the scaling factor, the stretched speech will have different rates depending on the rate of the input speech. Therefore, authors of this paper have proposed the method in which, as the control value of time-expansion, a desired ROS_d_ value is used. The value of the ROS_d_ is specified by the user. As a result of speech modification, stretched speech has the rate close to the ROS_d_ value. The signal processing procedure applied to this method is the same as in the algorithm B, but the current value of scaling factor is calculated for every signal frame separately, according to equations (2)(3)(4):

(2) $$\alpha_{\mathrm{cons}}\left(t\right)=\frac{\alpha \left(t\right)\cdot \Delta t}{\left(\eta -1\right)\Delta t_{\mathrm{vowel}}+\Delta t}$$

(3) $$\alpha \left(t\right)=\frac{ROS\left(t\right)}{ROS_{d}}$$

(4) $$\alpha_{\mathrm{vowel}}\left(t\right)=\eta \cdot \alpha_{\mathrm{cons}}\left(t\right)$$

where α_cons_(t) is the value of scaling factor for the current frame (provided a consonant was detected), α_vowel_(t) is the value of scaling factor for the current frame (provided a vowel was detected) , *Δt* is the time interval used for the ROS estimation (in the experiment, it was set to 1.5 s), Δt_vowel_ is the duration of the vowel in the estimation interval, *η* is the ratio between the scaling factor used for the vowels and the scaling factor used for consonants (in the experiment, it was equal to 1.7).

**Figure 2:**
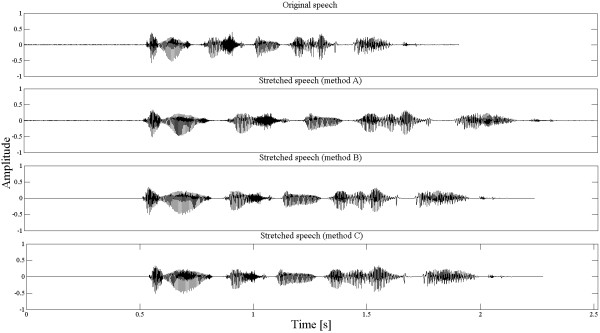
Example of speech stretched using methods A-C.

Examples of speech stretching obtained using the proposed methods are shown in Figure 2. In these examples, α_d_ was set to 1.5 (for method A and B) and ROS_d_ was equal to 3 vowels/s. These values of the scaling factor were also used during speech intelligibility tests described in Section 3. The choice of α_d_ value was based on the results presented by Nejime et al.[7]. He had shown that for α_d_ equal to 1.5, the highest improvement in speech comprehension could be achieved. The chosen value of ROS_d_ ensures the same ROS expansion as for the methods A and B.

An analysis of Figure 2 shows that the lowest difference in the duration of the stretched and the original speech is obtained using the method B. The method A produces the highest differences in the utterance duration. If not much redundancy is found in the input signal (using detectors), the signal can be time-expanded for a relatively long time and differences between the input and the output signal can drift towards infinity. To prevent such a situation, the TSM procedure is turned off after the difference between the input signal and output signal is higher than Δt_off_, and the unmodified speech is send to the output. This threshold is exceeded much often for the method A than for methods B and C and its value can be defined by the user. During the experiments, Δt_off_ was set to 3 seconds.

## Results

### Methodology

Evaluation of the proposed methods of TSM was performed employing the sentence intelligibility test (SIT). A word recognition test was not performed, because as it was shown by the Nejime [8], time expansion of speech has no impact on the intelligibility of separated words.

### Speech intelligibility test (SIT)

In case of SIT, 4 different types of speech were examined, i.e. the original speech and the speech stretched using three proposed TSM methods. As the speech material, Polish matrix test (PMT) [20] for elderly listeners and pediatric Polish matrix test (PPMT) for children were used. Usability of these tests for speech intelligibility measurements was examined and proved by the authors of the mentioned tests [21]. In both matrix tests, each sentence has the same grammatical structure. Sentences consist of 5 words for PMT and 3 words for PPMT. The procedure of sentence creation is the same as for the typical matrix test designed by Hagerman [22]: the list of words is fixed (to 50 words for PMT, and to 48 words for PPMT) and sentences are created by a random selection of words according to the sentence structure. This approach produced 100000 different sentences for PMT and 256 different sentences for PPMT (for more details, see papers [20,21,23]). The words necessary for both tests were recorded in a voice recording studio by a male speaker. All sets of words were recorded in three different average rates of speech measured in vowels/s, namely: 2.72, 4.88, 6.48 for PMT and 3.56, 6.43 and 7.58 for PPMT. Two highest rates for PPMT (ROS_mean_^1^ = 7.58 vowels/s, ROS_mean_^2^ = 6.43 vowels/s) and PMT (ROS_mean_^3^ = 6.48 vowels/s, ROS_mean_^4^ = 4.88 vowels/s) were used as the input signal during the experiment. In case of SIT, sentences were divided into two separate sets. The first set contained 40 sentences spoken with the highest speech rate (10 sentences for one type of algorithm: original, A, B, C), and the second set included 40 sentences with the second highest speech rate. During the test, each listener had to repeat words constituting the sentences. The word error rate (WER), as well as average improvement in speech intelligibility were measured. For WER calculation, the percentage of words repeated incorrectly was used, while the improvement of speech intelligibility, obtained for the proposed methods, was calculated as a difference between the WERs for the original and for the time-expanded speech.

### Time compressed speech test (TCST)

Additionally, each listener performed a time-compressed speech test (TCST) in order to obtain their individual 50% time-compressed speech threshold (TCT_50_) defined by Versfeld [24], as the alternative of SRT_50_ (Speech Reception Threshold) [23]. Speech material in this test was the same as in the SIT test. Since the rate of speech is artificially increased during the TCST, the average ROS of the input speech should be as low as possible to ensure a wide range of ROS values. Therefore, the average ROS of the input speech used for the test was equal to 3.56 vowels/s for the PPMT and 2.72 vowels/s for the PMT. It was observed that the chosen values of speech rate are perceived as a slow one. Originally, the TCT_50_ value represented a threshold, defined in syllables/s, for which 50% of the sentences in the test were correctly recognized by the listener. In our research, we have used a speech rate expressed as number of vowels/s. Thus, the speech rate defined by us is the derivation of the number of syllables/s.

The main difference between the test proposed by Versfeld [24] and a standard time-compressed speech test [25] is that in the standard test, its output provides the value of stretching factor which is independent from the rate of the input speech. Consequently, the results of this test for different speech materials cannot be compared with each other and they do not provide information about the ROS which is suitable for the listener. In turn, the results of the test proposed by Versfeld provide this kind of information, so it can be directly linked with intelligibility of time-expanded speech. The procedure related to the TCST test proposed by the Versfeld is as follows:

• each listener has to repeat 13 sentences

• the scaling factor is modified for each sentence according to the rules:

• if all words in the last sentence were repeated correctly, the value of scaling factor increases,

• otherwise the value of scaling factor decreases.

• TCT_50_ is calculated as a geometric average of the last 10 average rates of the sentences.

### Groups of listeners

For the elderly listeners, a tonal audiometry was performed in order to obtain their hearing level threshold. Listeners were using binaural headphones during the TCST and SIT and the signal level was set to a comfortable value. Each listener was asked at the beginning of test if the speech level is appropriate for them and if not, the level was adjusted. All listeners with hearing aids (HA) and cochlear implants (CI) were using their devices during the experiment.

The group of elderly people was examined during one session which lasted about 40 minutes. Tests for children were conducted in two separate sessions, because it was difficult for them to concentrate on the test for so long. Examination of children was divided into part one: TCST and SIT performed for the input speech with the average ROS equal to ROS_mean_^1^ (this part lasted about 20 minutes); and part two: SIT performed for the input speech with the average ROS equal to ROS_mean_^2^ (this part lasted about 12 minutes). The hearing thresholds were provided by the audiologist who performed this examination earlier.

In both groups of listeners, 17 volunteers were investigated. The average age of hearing impaired children was 9.35 years (9 females, 8 males), whereas the average age of elderly listeners was 80.76 years (11 females, 6 males).

### Experiments

The results obtained for both groups of listeners were analyzed in order to verify a hypothesis that for listeners with a low value of TCT_50_, time expansion of speech significantly improves the speech perception. At the beginning, the results of TCST were analyzed in order to divide listeners (the hearing impaired children, the elderly listeners) into subgroups containing listeners with the normal and the reduced hearing resolution. In Table 2, results obtained during the TCST are presented. It can be seen that, for the group of elderly listeners, the average value of TCT_50_ (4.81 vowels/s) is lower than for the hearing impaired children. The difference is mainly related to the differences occurring in the input speech material. For the children, three-word sentences were used during the experiment (PPMT) while for the adults, five-words sentences were used (PMT). Longer sentences are more difficult to remember and repeat. Therefore, authors have assumed that the reason of the differences is associated with the length of sentences.

**Table 2:** TCST results obtained for the hearing impaired children and the elderly listeners

**Listeners**	**TCT**_**50**_**[vowels/s]**		
	**Average value**	**Standard deviation**	**95% confidence interval**
Hearing impaired children	6.33	1.21	5.7/6.96
Elderly listeners	4.81	1.59	3.99/5.63

**Figure 3:**
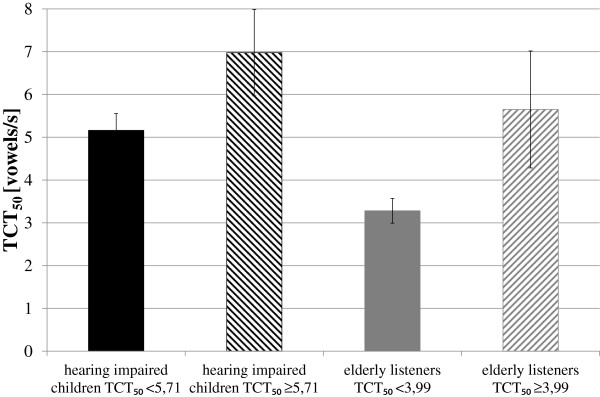
**TCT**_**50**_**thresholds for the hearing impaired children and the elderly listeners.**

Both groups of listeners were divided into subgroups based on Versfeld’s assumptions [24], i.e. if a person has TCT_50_ value lower than the 95% confidence interval value, then his hearing resolution is reduced. Two TCT_50_ thresholds were used. For the group of hearing impaired children, TCT_50_ threshold was equal to 5.71 vowels/s and for the elderly listeners it was 3.99 vowels/s. In Figure 3, average values of TCT_50_ calculated for all subgroups of listeners are presented. The subgroups of hearing impaired children and elderly people with the reduced hearing resolution contained 6 listeners and in the subgroups of listeners with the normal hearing resolution, 11 hearing impaired children and 11 elderly people were included.

Analysis of the SIT was performed separately for the group of the hearing impaired children and the elderly listeners. Statistical importance of the differences between mean values of the WER obtained for the original speech and the modified one was examined using an one-way repeated measures ANOVA (RM ANOVA) test and the Friedman’s test. For all analyses, a normal distribution of data was checked using the Shapiro-Wilk test and the hypothesis of the sphericity was verified using the Mauchly's test. The RM ANOVA test was performed only when both assumptions (normal distribution and sphericity) were met, otherwise the non-parametric Friedman test was used. For all tests, the significance level equal to 0.05 was assumed.

### Hearing impaired children

The hearing impaired children group contained 9 children with CI and 8 children with HA. The results of hearing tests obtained for this group of listeners were presented in Table 3 and Table 4. The hearing threshold for the group of children with HA was assessed using the tonal audiometry, and for the group of children with CI – using the ABR (Auditory Brainstem Response) test. Additionally, detailed results of the test are presented in Appendix A.

**Table 3:** **Results of hearing tests obtained for the hearing impaired children with reduced hearing resolution threshold (TCT**_**50**_ **< 5.71 vowels/s)**

**No**	**Age**	**Hearing thresholds [dB HL]**	**TCT**_**50**_**[vowels/s]**	**WER [%] ROS**_**mean**_^**1**^	**WER [%] ROS**_**mean**_^**2**^
1 (CI)	9	85^†^	5.04	23.33	58.33
2 (CI)	9	95^†^	5.34	26.67	53.33
3 (CI)	14	100^†^	5.01	36.67	66.67
4 (HA)	12	68.75^*^	4.53	0	62.50
5 (HA)	12	65^*^	5.39	13.33	66.67
6 (HA)	6	33.75^*^	5.66	6.67	29.17
average value	10.33	74.58	5.16	17.78	56.11
standard deviation	2.87	24,35	0.39	13.61	14.16

Meaning of the symbols near the results of hearing threshold is as follows: *- tonal audiometry results; † − ABR results.

**Table 4:** **Results of hearing tests obtained for the hearing impaired children with normal hearing resolution threshold (TCT**_**50**_ **≥ 5.71 vowels/s)**

**No**	**Age**	**Hearing thresholds [dB HL]**	**TCT**_**50**_**[vowels/s]**	**WER [%] ROS**_**mean**_^**1**^	**WER [%] ROS**_**mean**_^**2**^
1 (CI)	14	100^†^	5.92	10.00	20.83
2 (CI)	14	90^†^	5.84	3.33	62.50
3 (CI)	8	100^†^	6.54	23.33	58.33
4 (CI)	6	110^†^	6.56	6.67	41.67
5 (HA)	14	86.25^*^	6.10	23.33	37.50
6 (CI)	4	85^†^	7.61	10.00	37.50
7 (CI)	12	105^†^	6.57	13.33	20.83
8(HA)	4	35^*^	7.39	23.33	70.83
9 (HA)	6	26.25^*^	8.51	0	16.67
10 (HA)	7	44.37^*^	8.85	33.33	45.83
11 (HA)	8	55^*^	6.85	3.33	29.17
average value	8.82	76.08	6.98	13.63	40.15
standard deviation	3.97	30.21	1.01	10.69	17.99

Meaning of the symbols near the results of hearing threshold is as follows: *- tonal audiometry results; † − ABR results.

It can be seen that WER obtained for the speech spoken with the average ROS equal to ROS_mean_^2^ is much lower than for the average ROS_mean_^1^. This relation is valid for both subgroups of the hearing impaired children (the normal and the reduced hearing resolution). It may be related to the fact that the ROS_mean_^2^ value (6.43 vowels/s) is close to the average values of the TCT_50_ obtained for both subgroups of the hearing impaired children (the subgroup with reduced hearing resolution μ(TCT_50_) = 5.16 vowels/s and the subgroup with normal μ(TCT50) = 6.98 vowels/s). Furthermore, the ROS_mean_^1^ value (7.48 vowels/s) is higher than the average values of the TCT_50_ achieved in both subgroups of children. It should be also pointed out that the average WER obtained in two subgroups for the speech spoken with the ROS equal to ROS_mean_^1^ is similarly high (56.11% for TCT_50_ < 5.71; 40.15% for TCT_50_ ≥ 5.71) and for the ROS equal to ROS_mean_^2^, it is comparatively small (17.78%, 13.63%, respectively). Based on these observations, the following conclusion can be made: the hearing impaired children in both subgroups had comparable problems with comprehension of the unmodified speech.

**Figure 4:**
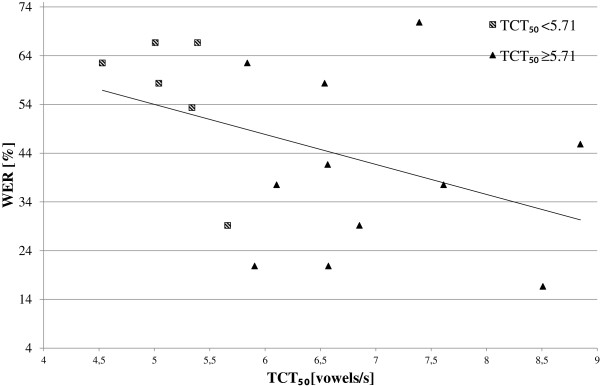
**Relationship between TCT**_**50**_**and WER for hearing impaired children.**

In order to verify if there is a correlation between the TCT_50_ and WER, a relationship between values of these measures are presented in Figure 4. The triangles represent test results obtained for the subgroup of hearing impaired children with the normal hearing resolution, and the squares indicate results for the subgroup of hearing impaired children with the reduced hearing resolution. The solid line shows the linear regression calculated for all hearing impaired children. The correlation coefficient between TCT_50_ and WER calculated for the subgroups of normal/reduced hearing resolution and for the whole group of hearing impaired children were: -0.09, -0.62, and −0.41, respectively. It can be seen that only for the subgroup of hearing impaired children with reduced hearing resolution, linear correlation is noticeable. Negative correlation value reflects the reverse relation. Therefore, the following conclusion can be made: the lower TCT_50_ was achieved in TCST, the higher WER value will be obtained in the SIT test for the high rate unmodified speech.

**Figure 5:**
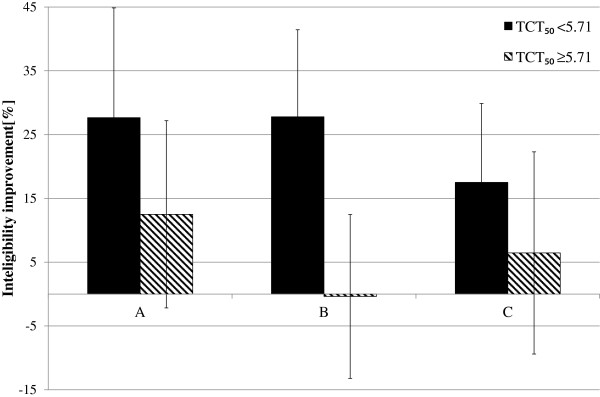
**Average improvement in WER for the input speech with average rate equal to ROS**_**mean**_^**1**^.

**Figure 6:**
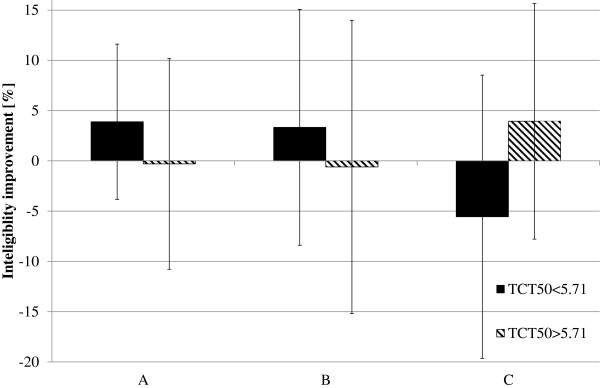
**Average improvement in WER for the input speech with average rate equal to ROS**_**mean**_^**2**^.

The influence of the proposed TSM methods on speech intelligibility was analyzed separately for two subgroups. The average values of intelligibility improvements are presented in Figure 5 (for the input speech spoken with the average rate of ROS_mean_^1^) and in Figure 6 (for the input speech spoken with the average rate of ROS_mean_^2^).

A significant improvement in the intelligibility can be mostly seen for the input speech spoken with average ROS equals to ROS_mean_^1^ (Figure 5) and speech modification algorithms A and C (from 6.43% for the children with normal hearing resolution and algorithm C to 27.63% for the algorithm A and children with the reduced hearing resolution). For the speech modified using the algorithm B, only the subgroup of children with reduced hearing resolution shows high improvement in speech intelligibility (27.77%).

To verify if the differences of the average WER values are statistically important, appropriate analyses were performed (separately for the input speech of ROS_mean_^1^ and ROS_mean_^2^). For the input ROS equal to ROS_mean_^1^, the results of SIT obtained by the subgroup of children with TCT_50_ < 5.71 vowels/s did not show the normal distribution. Therefore, for these data, Friedman’s non-parametric test was applied. The statistic value of the test was equal to χ^2^(3) = 113.5. Since the number of listeners in this subgroup was low (6 persons), in order to increase the reliability of the result, the p-value was not calculated but the test statistic value was compared with the suitable critical value read from the tables (χ^2^(3)_cv_ = 76). Hence, the critical test value is lower than the obtained Friedman’s statistic value, the differences between the WERs obtained for the unmodified speech and the speech modified using algorithm A-C are statistically important. In order to check which algorithm causes this situation, a post hoc non-parametric test equivalent to the Least Significant Difference Fisher’s test was performed. For the pairs of unmodified speech and speech modified using algorithms from A to C, the following statistical values were obtained: 12.5 (A), 13.5 (B), and 8.0 (C). These results were compared with the critical value equal to 6.2. Since all the statistics values were higher than the critical value for all the proposed algorithms, differences between the WER obtained for the unmodified and modified speech are statistically important.

For the same speech rate and the subgroup of children with TCT_50_ ≥ 5.71 vowels/s, the normal distribution was confirmed (for all algorithms) and the assumption of sphericity was met. For these reasons, RM ANOVA was calculated for these data. The following results were obtained: F(3,30) = 3.44 and p = 0.03. Since the achieved p value is lower than the assumed significance level in at least one pair, the differences between average values of WER are statistically important. Additionally, the post hoc LSD test shows that only differences in average WER between the pairs ‘original-method A’ (t(10) = −2.83; p = 0.02) and ‘method A- method B’ (t(10) = 2.54); p = 0.03) are statistically important. This results shows that for the subgroup of hearing impaired children with the normal hearing resolution, method A gives statistically important improvement of in speech intelligibility.

**Figure 7:**
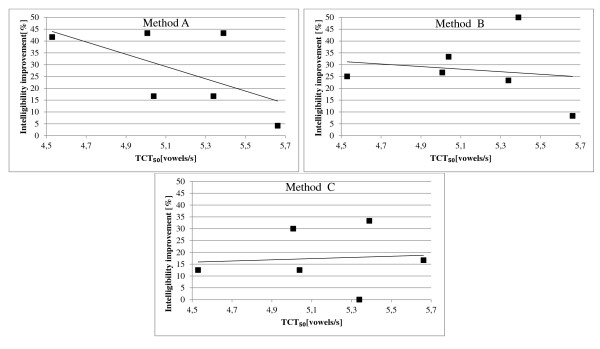
**Relationship between the TCT**_**50 **_**values and the improvement in speech comprehension obtained for the subgroup of children with low hearing resolution.**

**Figure 8:**
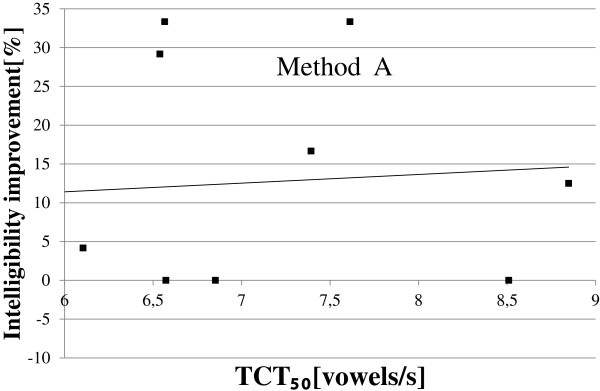
**Relationship between the TCT**_**50 **_**values and the improvement in speech comprehension obtained for the subgroup of children with normal hearing resolution.**

Additionally, the correlation coefficient was calculated to validate if there is a linear relationship between the TCT_50_ for the subgroup of hearing impaired children with the reduced hearing resolutions and the improvement in speech ineligibility for the time-expanded speech while using methods A-C, as well as for the subgroup of hearing impaired children with normal hearing resolution and the improvement in speech ineligibility for the time-expanded speech while using method A. The correlation coefficient obtained for the first subgroup for methods A-C were equal to −0.59 (A), -0.16(B), and 0.08 (C). Only for the method A there is a significant linear correlation between these values. For the second subgroup and the method A, the correlation coefficient was equal to 0.07. Therefore, there is no linear correlation between these values. In Figure 7 and in Figure 8, these relationships are presented and the linear regression curve was added to illustrate the correlation values.

In case of the speech spoken with ROS_mean_^2^, improvement in speech ineligibility was observed only for the subgroup of children with reduced hearing resolution and methods A-B (3.89% and 3.34%, respectively) and for the subgroup of children with normal hearing resolution and method C (3.94%). In other cases, a slight decrease in WER was observed (from −5.55% to −0.3%). For the subgroup of children with reduced hearing resolution, RM ANOVA was calculated (data had normal distribution and assumption of sphericity was met). The results of the test show that there are no statistically important differences in WER between the analysed methods (F(3,15) = 1.51; p = 0.25). For the subgroup of children with normal hearing resolution, the Friedman’s test was performed (data were not normally distributed) and there was no statistically important difference in WER between the methods (χ^2^(3) = 0.30; p = 0.82). Therefore, none of the proposed methods affect the intelligibility of speech spoken with ROS equal to ROS_mean_^2^.

### Elderly listeners

The results of hearing tests performed for the group of elderly listeners are presented in Table 5 and Table 6 (detailed results of the test are presented in Appendix B.). Three subjects in this group were wearing hearing aid during the TCST and SIT tests. Similarly to the group of hearing impaired children, WER for the speech spoken with higher rate (ROS_mean_^3^ = 6.48 vowels/s) is higher than for the speech spoken with the lower rate (ROS_mean_^4^ = 4.88 vowels/s). However, for this group of listeners, the differences in WER between the subgroups of listeners with normal and reduced hearing resolution are significant (normal: 12.36%, 6.18%; reduced hearing resolution: 51.33%, 31.67%).

**Table 5:** **Results of hearing tests obtained for the elderly listeners with normal hearing resolution threshold (TCT**_**50**_ **< 3.99 vowels/s)**

**No**	**Age**	**Mean audiometric hearing thresholds [dB HL]**	**TCT**_**50**_**[vowels/s]**	**WER [%] ROS**_**mean**_^**3**^	**WER [%] ROS**_**mean**_^**4**^
1	83	56.87	3.22	22	28
2	87	68.75	3.08	34	28
3	85	63.75	3.50	48	54
4	91	70	2.93	56	78
5(HA)	89	73.75	3.73	22	66
6	86	55.62	3.23	8	54
average value	86.83	64.79	3.28	31.67	51.33
standard deviation	2.86	7.36	0.29	17.95	20.15

**Table 6:** **Results of hearing tests obtained for the elderly listeners with normal hearing resolution threshold (TCT**_**50**_ **≥ 3.99 vowels/s)**

**No**	**Age**	**Mean audiometric hearing thresholds [dB HL]**	**TCT50 [vowels/s]**	**WER [%] ROS**_**mean**_^**3**^	**WER [%] ROS**_**mean**_^**4**^
1 (HA)	86	58.75	4.25	10	14
2 (HA)	77	38.75	5.39	10	20
3	82	66.25	4.17	8	28
4	74	30.62	6.52	8	18
5	83	23.12	5.02	10	22
6	76	40	4.23	8	10
7	89	45.62	6.38	6	12
8	76	28.75	7.26	0	0
9	81	42.5	4.41	4	6
10	64	22.5	8.12	0	0
11	64	18.12	6.38	4	6
average value	77.45	37.73	5.65	6.18	12.36
standard deviation	8.05	15.19	1.37	3.73	9.07

**Figure 9:**
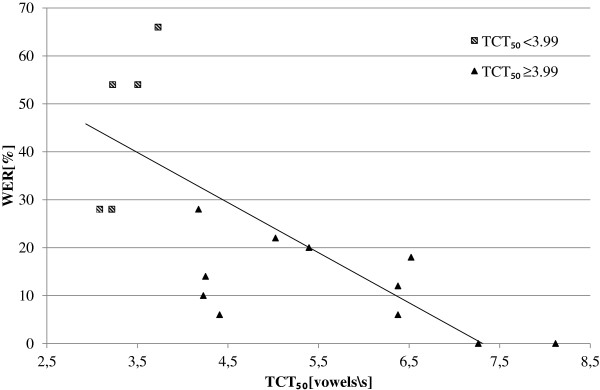
**Relationship between TCT**_**50 **_**and WER for the elderly listeners.**

Based on the obtained results, it was investigated if there is a linear correlation between the TCT_50_ and WER. In Figure 9, a relationship between TCT_50_ and WER was presented. The triangles represent the results obtained by the subgroup of elderly listeners with normal hearing resolution and the squares – the results achieved by the subgroup of subjects with reduced hearing resolution. The solid line shows the linear regression calculated for all elderly listeners. The Pearson correlation coefficients calculated for both subgroups (normal/reduced hearing resolution) and for all elderly subjects were equal to: -0.71, 0.12 and −0.58, respectively. Only for the subgroup of subjects with normal hearing resolution, the linear correlation could be found.

**Figure 10:**
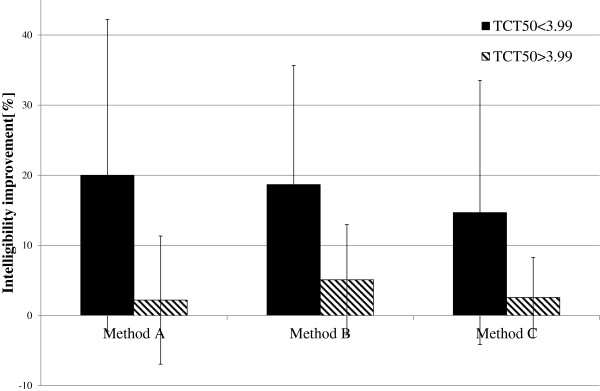
**Average improvement in WER for input speech with average rate equal to ROS**_**mean**_^**3**^.

**Figure 11:**
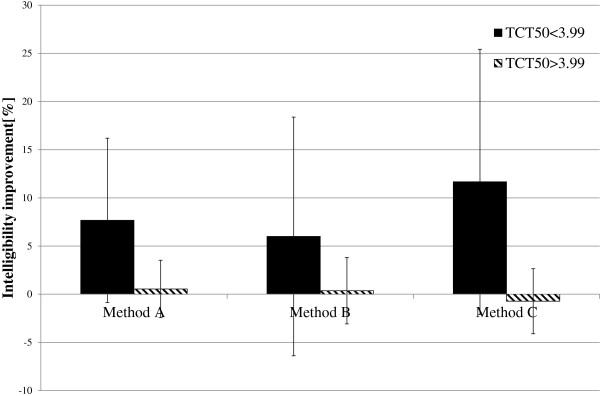
**Average improvement in WER for input speech with average rate equal to ROS**_**mean**_^**4**^.

Influence of the proposed time-expansion method was analysed separately for two different input speech rates (ROS_mean_^3^ and ROS_mean_^4^) and for two subgroups (reduced/normal hearing resolution). In Figure 10 and Figure 11, the average improvement in speech intelligibility obtained by those subgroups of listeners are presented. The highest improvement could be observed for the listeners with reduced hearing resolution. For these subjects, the trend is noticeable for two speech rates (for ROS_mean_^3^_:_ improvement from 14.66% to 20% and for ROS_mean_^4^_:_ from 6% to 11.66%). For the subgroups of listeners with reduced hearing resolution, a small improvement was observed only for speech spoken with the ROS equal to ROS_mean_^3^ (from 2.18% to 5.09%) and for the subgroup of subjects with normal hearing resolution, the improvement is negligible (from −0,72% to 0,54%). For the speech spoken with the ROS equal to ROS_mean_^3^ in the subgroup of listeners with the reduced hearing resolution, both conditions of the RM ANOVA test were met. The test results prove that there are statistically important differences in WER between the proposed methods (F(3,15) = 4.36, p = 0.021). The post hoc LSD test indicates that these differences are significant only for the method B (t(5) = −2.68, p = 0.043). For the subgroup of listeners with normal hearing resolution, the RM ANOVA test did not confirm that the differences between WER values are statistically important (F(3,30) = 1.25, p = 0.3). For the speech spoken with ROS equal to ROS_mean_^4^, and for both subgroups of elderly listeners, the RM ANOVA test shows that the differences between the obtained WER values are not statistically important (F(3,15) = 2.62, p = 0.089; F(3,30) = 0.48, p = 0.69).

**Figure 12:**
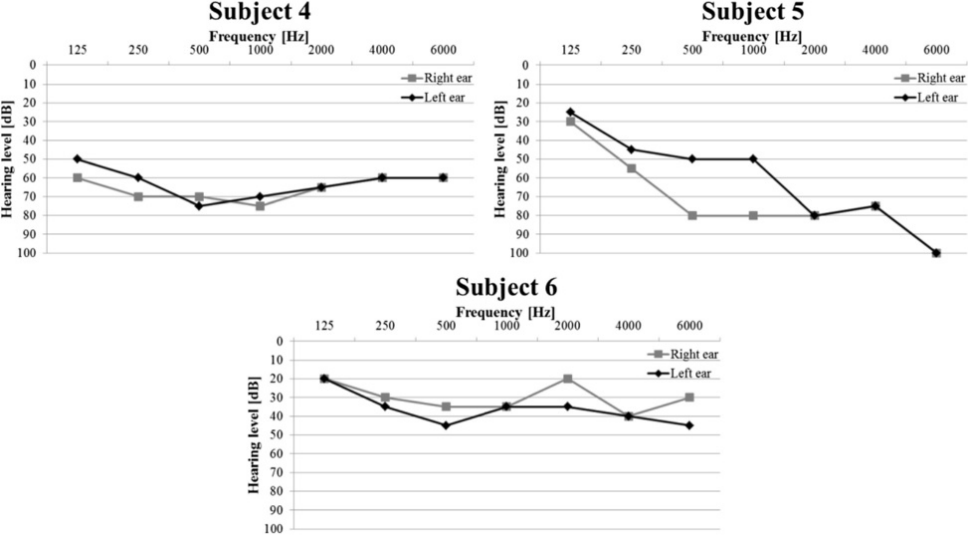
Audiometric test results for the group of hearing impaired children with reduced hearing resolution.

Since the observed improvement in speech comprehension was statistically important only for the method B, a relationship between the TCT_50_ and the intelligibility improvement was analysed only for these data. In Figure 12, this relationship is presented. The calculated Pearson correlation coefficient was equal to 0.58 and its positive value indicates that the improvement in speech comprehension (provided by the method B) is higher when the hearing resolution of listener increases. This observation is surprising because the inverse relationship was expected (the higher hearing resolution, the lower improvement of speech comprehension). This phenomenon may be caused by the fact that in the group of elderly listeners with reduced hearing resolution, only one subject was using HA and the hearing losses of all the listeners in this subgroup were also significant (see Table 5). Consequently, two hearing impairments overlap here and cause the difficulties in speech comprehension.

## Discussion

The speech intelligibility test performed in two groups of listeners (the hearing impaired children and the elderly listeners) have shown that there are differences in speech comprehension in case of time scale modified speech in comparison to the original one. These differences are significant only if a very fast speech is used as the input signal for the test (ROS_mean_^1^_,_ ROS_mean_^3^). For the hearing impaired children with reduced hearing resolution, all the proposed methods gave statistically important improvement. For the group of elderly listeners, only the method B produces statistically important improvement of speech comprehension. The importance of this improvement was proved in the group of listeners with very low hearing resolution (TCT_50_ < 3.99 vowels/s). These results are similar to those presented by Nejime et al. [9], who verified their method for listeners with reduced hearing resolution (measured using RGDT - Random Gap Detection Test). Nejime et al. showed the importance of speech modification using their method, which stretches only the high energy parts of speech. Nejime did not found a clear relationship between the hearing impairment or hearing resolution and the results of WER. This relationship was shown in our research. First, for the subgroup of hearing impaired children with reduced hearing resolution, a significant correlation between TCT_50_ and WER was observed. Second, a not so high but significant correlation was found for the relation between TCT_50_ and the improvement (in subgroups with deficit in hearing resolution) of intelligibility of speech stretched using the method B for elderly listeners and the method A for the hearing impaired children.

For the speech spoken with lower rates (ROS_mean_^2^, ROS_mean_^4^), the obtained results are consistent with those presented by Uchanski et al. [11]. There are no statistically important differences in speech intelligibility between the original speech and the time-expanded one.

## Conclusions

Three methods for real-time speech stretching were proposed and verified experimentally. It was proved that the method B significantly improves speech comprehension in hearing impaired children, as well as in elderly people with deficit in the hearing resolution. In turn, the proposed non-uniform real-time speech stretching method A brings satisfying results not only for the hearing impaired children with low value of TCT_50_, but also for children with normal hearing resolution. The presented results are in a good accordance with the state-of-the-art results and extend them with the introduction of analysis of the impact of the input speech rate on the relation between the TSM and speech intelligibility. Another novelty is the usage of TCT_50_ as a measure of the hearing resolution deficit. It was shown that this parameter correlates with the improvement achieved by employing time-expanded speech.

## Appendix A

**Figure 13:**
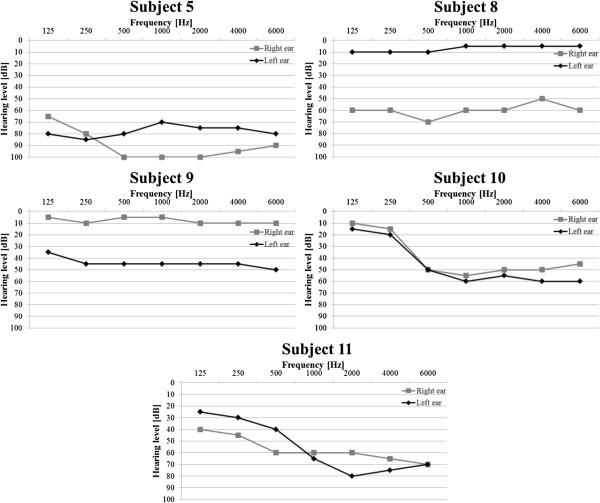
Audiometric test results for the group of hearing impaired children with normal hearing resolution.

**Figure 14:**
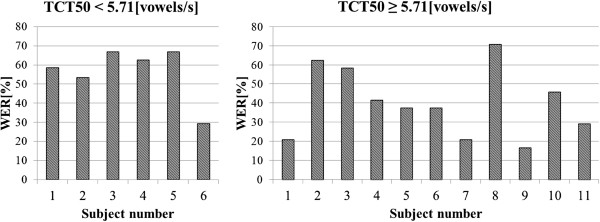
Average WER achieved by the hearing impaired children for input speech with ROS equal to ROS_mean_^1^.

**Figure 15:**
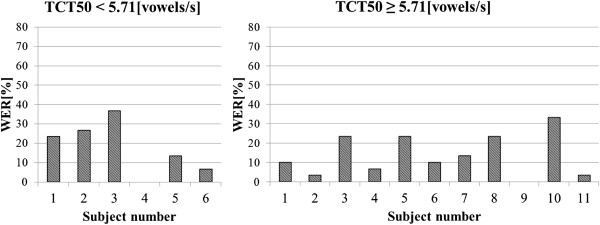
Average WER achieved by the hearing impaired children for input speech with ROS equal to ROS_mean_^2^.

In Figures 12 and 13 results of the audiometric test obtained for the group of hearing impaired children were presented. In Figure 14 and 15 average WER achieved by this group of liseteners for the input speech with ROS equal to ROS_mean_^1^ and ROS_mean_^2^ was shown.

### Appendix B

**Figure 16:**
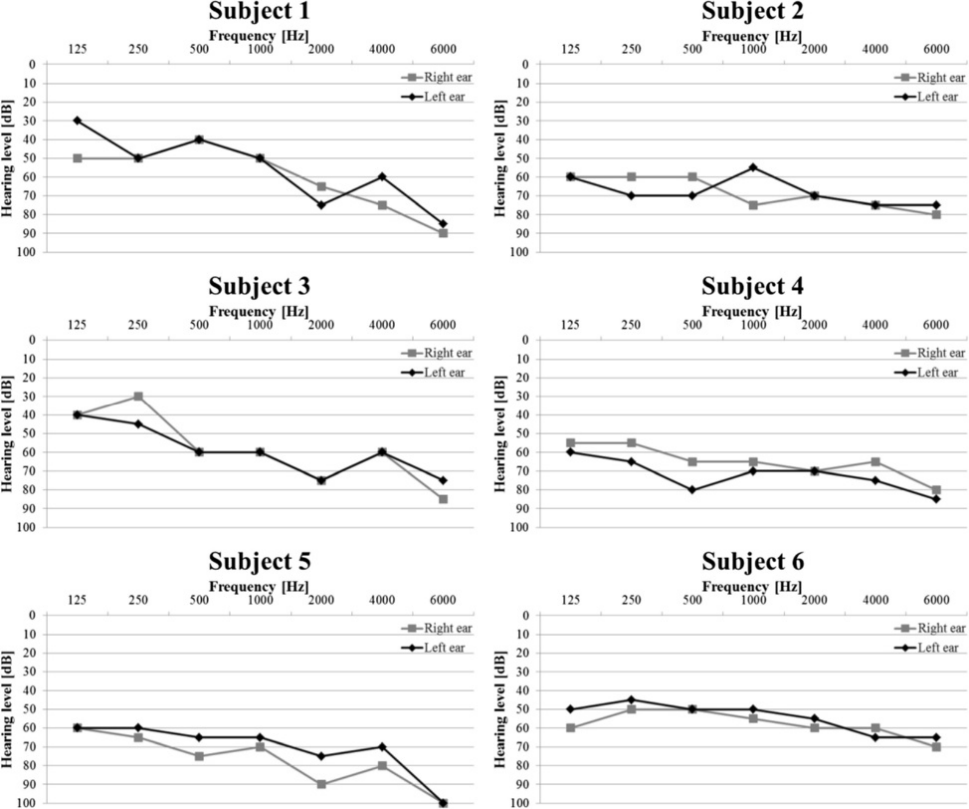
Audiometric test results for the group of elderly listeners with reduced hearing resolution.

**Figure 17:**
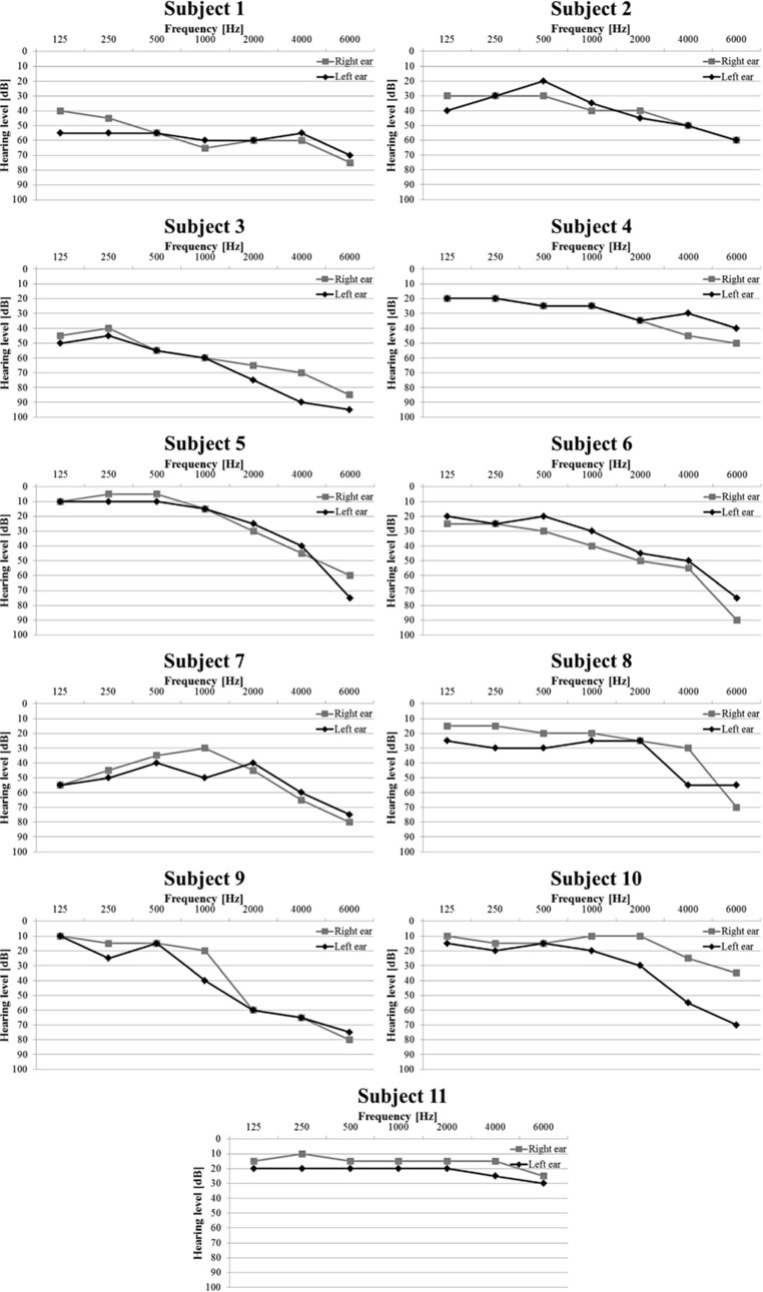
Audiometric test results for the group of elderly listeners with reduced hearing resolution.

**Figure 18:**
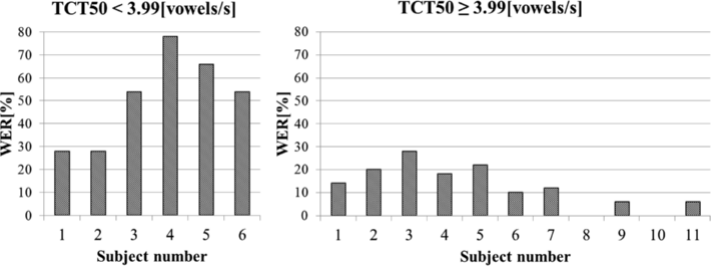
Average WER achieved by the elderly listeners for input speech with ROS equal to ROS_mean_^3^.

**Figure 19:**
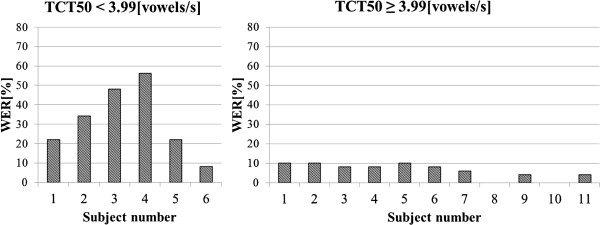
Average WER achieved by the elderly listeners for input speech with ROS equal to ROS_mean_^4^.

In Figure 16 and 17 results of the audiometric test obtained for the group of elderly listeners were presented. In Figure 18 and 19 average WER achieved by this group of listeners for the input speech with ROS equal to ROS_mean_^3^ and ROS_mean_^4^ was shown.

## Abbreviations

TSM: Time Scale Modification; LLI: Language Learning Impaired; CAPD: Central Auditory Processing Disorders; VAD: Voice Activity Detection; SOLA: Synchronous Overlap and Add; ROS: Rate of Speech; SIR: Sentence Intelligibility Test; PMT: Polish Matrix Test; PPMT: Pediatric Polish Matrix Test; WER: Word Error Rate; TCST: Time-Compressed Speech Test; SRT_50_: Speech Reception Threshold; TCT_50_: 50% time-compressed speech threshold; ANOVA: Analysis Of Variance; RM: ANOVA: Repeated Measures ANOVA; CI: Cochlear Implant; HA: hearing aid; LSD: Least Significant Difference; RGDT: Random Gap Detection Test.

## Competing interests

The authors declare that they have no competing interests.

## Authors’ contributions

Conceived and designed the experiments: AK, AC. Performed the experiments: AK. Analyzed the data: AK. Wrote the paper: AK, AC. Revised the paper: AK, AC. All authors read and approved the final manuscript.
